# Successful Nonsurgical Management of Norovirus-Associated Necrotizing Enterocolitis in a Preterm Infant: A Case Report

**DOI:** 10.3390/children13101406

**Published:** 2026-10-14

**Authors:** Linda Sessa, Lucilla Martini, Marta Tedesco, Alessandro Ferrazza, Matteo Stefanini, Nicoletta Menzella, Francesca Priolo, Giovanni Vento, Simonetta Costa

**Affiliations:** 1 Neonatology Unit, Ospedale Policlinico Casilino, 00169 Rome, Italy; lumartini@policlinicocasilino.it (L.M.); mtedesco@policlinicocasilino.it (M.T.); scosta@policlinicocasilino.it (S.C.); 2 Radiodiagnostic Unit, Ospedale Policlinico Casilino, 00169 Rome, Italy; aferrazza@policlinicocasilino.it (A.F.); mstefanini@policlinicocasilino.it (M.S.); 3 Department of Woman and Child Health and Public Health, Fondazione Policlinico Universitario Agostino Gemelli, IRCCS—Catholic University of Sacred Heart, 00168 Rome, Italy; nicoletta.menzella@guest.policlinicogemelli.it (N.M.); francesca.priolo@policlinicogemelli.it (F.P.); giovanni.vento@unicatt.it (G.V.)

**Keywords:** intestinal pneumatosis, intravenous immunoglobulin, necrotizing enterocolitis, norovirus infection, preterm infant

## Abstract

**Highlights:**

**What are the main findings?**

- A preterm neonate with stool RT-PCR-confirmed Norovirus infection developed Bell stage IIA necrotizing enterocolitis with intestinal pneumatosis.
- Intestinal pneumatosis resolved with conservative management, without perforation or long-term gastrointestinal sequelae.

**What are the implications of the main findings?**

- Norovirus-associated intestinal disease in preterm neonates may have a favourable course despite radiological evidence of intestinal pneumatosis.
- Conservative management may be a reasonable approach in selected clinically stable neonates, provided that close clinical and radiological monitoring is ensured.

**Abstract:**

Norovirus infection has been increasingly reported in association with necrotizing enterocolitis (NEC) in preterm neonates, although the clinical spectrum remains poorly defined. We report the case of a 29-week preterm male infant who developed abdominal distension on day 35 of life, with a sepsis-like clinical presentation. Stool reverse transcriptase polymerase chain reaction confirmed Norovirus infection. Serial abdominal radiographs showed intestinal pneumatosis from day 2 of illness, consistent with Bell stage IIA NEC. The pneumatosis persisted for three days and completely resolved by day 5, without perforation or clinical deterioration. The infant was managed conservatively with bowel rest, parenteral nutrition, broad-spectrum antibiotics, and mechanical ventilation for 7 days. IVIG (500 mg/kg/day for 4 days) was administered for clinically suspected capillary leak syndrome. Enteral feeding was restarted after 8 days, and the infant was discharged at 71 days of life without gastrointestinal complications. This case highlights the heterogeneous clinical spectrum of Norovirus-associated intestinal disease and suggests that conservative management may be successful in selected clinically stable preterm infants with NEC and intestinal pneumatosis.

## 1. Introduction

Necrotizing enterocolitis (NEC) is a devastating gastrointestinal emergency affecting approximately 5–10% of very preterm or very low-birth-weight neonates, with mortality rates of approximately 20–30% [1,2].

Despite decades of research and advances in neonatal care, NEC remains a leading cause of mortality and morbidity in neonatal intensive care units worldwide, with mortality rates approaching 20% overall and 30–50% in advanced cases [3].

The risk factors for NEC include extreme prematurity, extremely low birth weight, intrauterine growth restriction with absent or reversed end-diastolic flow on umbilical artery Doppler studies, formula feeding, sepsis, congenital heart disease and blood transfusions. The disease spectrum ranges from gastrointestinal symptoms to catastrophic illness characterized by circulatory compromise, respiratory and metabolic acidosis, disseminated intravascular coagulopathy and multiorgan system failure [4].

The pathogenesis of NEC is multifactorial and involves the interaction of intestinal immaturity, impaired intestinal barrier function, dysregulation of the intestinal immune response, alterations of the gut microbiota, and environmental factors. In preterm infants, the immature intestinal barrier and dysregulated inflammatory response may increase susceptibility to intestinal injury and facilitate the progression of inflammation. Increasing evidence suggests that infectious agents, including viral pathogens, may contribute to this complex pathophysiological process [4].

The Bell staging system [5], first proposed in 1978 and subsequently modified by Walsh and Kliegman [6], remains the most commonly used method to assess NEC severity.

Emerging evidence demonstrated a significant association between viral infections and NEC development in preterm neonates. Among specific viruses, Norovirus showed the strongest association with NEC [7]. The reported cases concerned premature infants who showed intestinal pneumatosis requiring laparotomy with colectomy and stoma creation. In many of the infants described, laparotomy showed intestinal stenotic areas.

We report the favourable clinical and radiological evolution of NEC in a preterm neonate with stool RT-PCR-confirmed Norovirus infection.

## 2. Case Presentation

The baby was a 29 weeks’ gestation male infant who experienced sudden onset of abdominal distension on the 35th day of life with worsening of general condition and sepsis-like clinical appearance. Clinically, the infant presented with marked abdominal distension and meteorism, with a markedly globose and poorly compressible abdomen, absent bowel sounds and mild abdominal tenderness. Minimal bilious gastric residual via the nasogastric tube was documented (<2 mL/kg). Laboratory investigations revealed leukopenia with severe neutropenia together with elevated inflammatory markers and anaemia requiring red blood cell transfusion. No thrombocytopenia or metabolic acidosis was documented. Blood, urine, and cerebrospinal fluid cultures were negative for bacteria. During the acute phase, there were no bowel movements apart from the initial evacuation where the presence of Norovirus was confirmed by reverse transcriptase polymerase chain reaction (RT-PCR). The main laboratory findings and vital signs during the acute phase of illness are summarized in Table 1.

**Figure 1:**
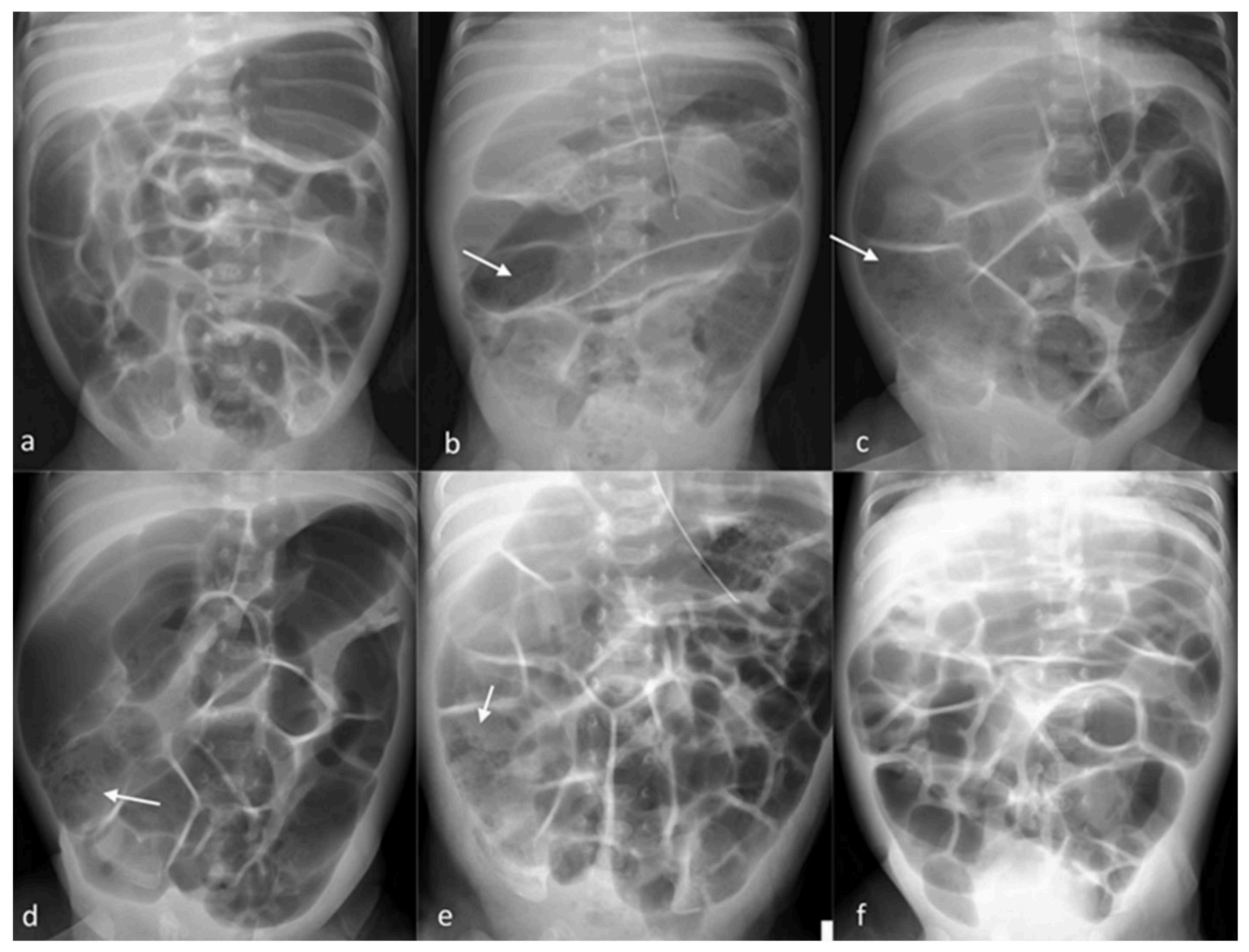
The radiological evolution of intestinal pneumatosis. Distension of intestinal loops, with a diameter greater than the width of the L5 vertebra, appeared at the onset of gastrointestinal symptoms (**a**). “Soap bubbles” appearance (arrows) emerged after two days from the onset of symptoms (**b**) and persisted for three days into treatment for necrotising enterocolitis (**c**–**e**). Pneumatosis was no longer visible after five days from the onset of symptoms (**f**).

The neonate was maintained for 7 days under mechanical ventilation to minimize gastrointestinal air insufflation in the setting of marked abdominal distension. Empirical antibiotic therapy with intravenous vancomycin and meropenem was initiated from the first day of symptom onset. A central venous catheter was placed for the administration of total parenteral nutrition. Serial abdominal plain radiographs were performed: at the beginning, we observed only distension of bowel loops (Figure 1a). Intestinal “soap bubbles” pneumatosis appeared on the second day of illness (Figure 1b) and persisted for three days (Figure 1c–e).

On the second day of illness, the infant developed generalized pitting edema, associated with a significant increase in body weight and preserved renal function. Due to the clinical diagnosis of capillary leak syndrome, treatment with intravenous immunoglobulin (IVIG) was started at a dose of 500 mg/kg/day for 4 days.

Considering the presence of intestinal pneumatosis on serial abdominal radiographs, in the absence of ascites, together with marked abdominal distension and meteorism, a markedly globose and poorly compressible abdomen, and absent bowel sounds, we assigned a Bell stage IIA NEC during the acute phase of the illness. No thrombocytopenia or metabolic acidosis was documented.

On the fifth day, pneumatosis was no longer visible (Figure 1f). No free air has ever been detected.

After seven days, the patient was transitioned to non-invasive ventilation, which was discontinued after 7 days, on the 49th day of life, because of satisfactory respiratory function. Antibiotic therapy was discontinued after 7 days following normalization of inflammatory markers and clinical improvement.

After eight days, the infant began to have spontaneous bowel movements and to feed. He was discharged at 71 days of life without abdominal complications due to NEC.

A detailed timeline of the main clinical, radiological, and therapeutic events from the onset of symptoms to discharge is provided in Table 2.

The baby is currently one year old and is in good clinical condition, shows adequate growth, and has not experienced any gastrointestinal symptoms.

## 3. Discussion

We reported the favourable evolution of NEC in a preterm infant, which did not reach the advanced stages characterized by intestinal perforation and which resolved without long-term gastrointestinal sequelae. This clinical course was different from that described so far in the literature in neonates with Norovirus infections [8,9].

Multiple outbreaks of NEC associated with Norovirus have been documented in neonatal intensive care units. Beyond the epidemiological association, several mechanisms may potentially explain a contribution of Norovirus infection to intestinal injury in preterm infants. The immature intestinal barrier of preterm neonates may increase susceptibility to viral-induced epithelial injury and facilitate the interaction between luminal pathogens and the intestinal immune system. Norovirus infection may also promote local inflammatory responses and alterations in intestinal barrier function, potentially amplifying an already dysregulated inflammatory environment in the premature intestine. In this context, Norovirus infection may act as an additional inflammatory or environmental trigger in a multifactorial process rather than as an independent cause of NEC. However, the specific mechanisms linking Norovirus infection to NEC remain incompletely understood and cannot be established from the available clinical evidence [7].

A 1998 outbreak in New York involved eight cases of NEC with two deaths. In this cohort of neonates, six stool samples were available for analysis and Norovirus particles were identified in 4 of 6 specimens. Of the eight reported cases, seven demonstrated intestinal pneumatosis on abdominal radiography. Persistent dilated bowel loops and portal venous gas were reported in two cases, while abdominal distension was present in four cases. No intestinal perforation was reported, but, except for the two fatal cases, the clinical outcomes and long-term evolution of the remaining patients were not described. The severity of NEC according to Bell’s staging criteria was not specified, nor were the causes of death, including whether they were directly related to intestinal complications. Furthermore, no detailed information was provided regarding the therapeutic management and clinical interventions [10].

Armbrust et al. described another outbreak of eleven cases of low-birth-weight premature infants with suggestive symptoms of viral infection. Norovirus were detected in stools in eight cases. Seven infants developed abdominal distension, and two developed NEC. In the first case with confirmed Norovirus infection, NEC stage IIB according to Bell’s criteria occurred and required surgical intervention with hemicolectomy and ileostomy. The second infant presented colitis requiring stoma formation, capillary leak syndrome, and multiorgan failure, resulting in death [8]. Interestingly, among infants with Norovirus infections described in the literature, only this infant presented capillary leak syndrome as a complication of the infection, as in our patient. No details of the therapeutic management of capillary leak syndrome were reported for this neonate in the Armbrust et al. case series. In our infant, IVIG was administered as capillary leak syndrome therapy [11,12]. Given the absence of specific antiviral therapies for Norovirus infection and the available literature supporting the use of IVIG in severe neonatal Enterovirus infections, IVIG was administered in our patient based on the reported immunomodulatory and virus-neutralizing properties of immunoglobulins [13,14]. Available evidence on the use of IVIG in neonatal invasive Enterovirus infections is mainly derived from case series and observational studies, and no definitive conclusions regarding its efficacy can be drawn in the absence of randomized clinical trials [15,16,17,18,19].

In a cohort of 34 infants with viral enteric infections admitted to a neonatal intensive care unit in Germany, Norovirus was detected in eight patients. The predominant clinical signs were abdominal distension, diarrhoea, and apnoea. Four infants presented with bloody stools, and only one showed an acute inflammatory response with elevated C-reactive protein and lactate, suggesting stage IB NEC. In this infant, enteral nutrition was discontinued and empiric antibiotic therapy was administered. In these eight reported cases, abdominal symptoms did not progress beyond Bell’s stage 1 and, therefore, did not overcome the suspicion of NEC [20].

Finally, Pelizzo et al. described a case series of three preterm infants with Norovirus-associated isolated colon ischemia from a NICU in Italy. The first case presented abdominal distension and radiological evidence of intestinal pneumatosis. Four days after symptom onset, the development of free intraperitoneal air led to surgical intervention, consisting of colectomy with ileostomy formation. Intraoperative findings revealed areas of intestinal stenosis associated with necrosis and intestinal perforation. The second and third cases presented with haematochezia and abdominal distension; intestinal pneumatosis was observed in the third case. These two patients underwent exploratory laparotomy, which revealed stenotic intestinal segments, requiring subsequent ileostomy formation. In all cases, enteral feeding was discontinued, and antibiotic therapy was administered. Therefore, Pellizzo et al. suggested early surgical management with ileostomy once colonic pneumatosis is identified on abdominal radiography [9]. These findings contrast with our reported infant, in which the clinical course was favourable with conservative management, despite the presence of intestinal pneumatosis which persisted for three days and without the need for surgical intervention. Our infant developed abdominal distension and radiographic evidence of pneumatosis intestinalis in the setting of RT-PCR-confirmed Norovirus infection, leading to the diagnosis of confirmed NEC. However, the abdominal clinical course remained stable, without progression to intestinal perforation. Serial abdominal imaging demonstrated gradual resolution of pneumatosis under conservative management, including bowel rest and supportive care. It is difficult to define exactly what influenced the favourable course of NEC in our newborn. Since NEC is a condition that recognizes an inflammatory pathogenesis, we can hypothesize that IVIG may provide the neutralization of enteral and circulating viral particles, modulating the systemic and intestinal inflammatory response.

## 4. Conclusions

Our report further supports the concept that Norovirus-related intestinal pathology in neonates encompasses a broad clinical spectrum, ranging from self-limiting forms of pneumatosis intestinalis to fulminant, life-threatening disease. Our case suggested that intestinal pneumatosis associated with Norovirus infection could resolve completely and without sequelae with conservative management in the absence of clinical deterioration or abdominal perforation.

## Figures and Tables

**Table 1:** Laboratory findings and vital signs during the acute phase of illness.

Parameter	Measured Value	Reference Range
**Vital sign**		
Heart rate	155 beats/min	100–180 beats/min
Peripheral oxygen saturation	97%	≥90%
**Hematological parameters**		
White blood cell count	3.37 × 10^3^/μL	5.00–17.00 × 10^3^/μL
Absolute neutrophil count	0.61 × 10^3^/μL	1.00–12.00 × 10^3^/μL
Haemoglobin	11.6 g/dL	9.0–15.0 g/dL
Haematocrit	33.2%	27.0–44.0%
Platelet count	387 × 10^3^/μL	220–500 × 10^3^/μL
**Inflammatory markers**		
C-reactive protein	77.6 mg/L	0–5 mg/L
**Acid-base status**		
pH	7.37	7.35–7.45
Base excess	1.9	−2–+3 mmol/L
Lactate	0.9	0.5–2.2 mmol/L
**Serum electrolytes**		
Sodium	135 mEq/L	136–145 mEq/L
Potassium	4.71 mEq/L	3.50–5.10 mEq/L
Chloride	103 mEq/L	98–107 mEq/L
Total calcium	9.3 mg/dL	8.4–10.6 mg/dL
**Microbiological investigations**		
Blood culture	Negative	Negative
Urine culture	Negative	Negative
Cerebrospinal fluid culture	Negative	Negative
Stool RT-PCR for Norovirus	Positive	Negative
Cerebrospinal fluid RT-PCR for Norovirus	Negative	Negative

Legend: RT-PCR, reverse transcriptase polymerase chain reaction.

**Table 2:** Timeline of the main clinical, radiological, and therapeutic events during the clinical course.

Time	Clinical Event
Day 35 of life	Acute abdominal distension and sepsis-like clinical deterioration
Day 1	Bowel rest, TPN, broad-spectrum antibiotics and mechanical ventilation initiated
Day 2	Intestinal pneumatosis detected; Bell stage IIA NEC assigned; capillary leak syndrome developed; IVIG started
Days 2–4 Intestinal pneumatosis persisted	
Day 5	Complete radiological resolution of pneumatosis
Day 7	Mechanical ventilation discontinued; transition to non-invasive ventilation
Day 8	Spontaneous bowel movements and enteral feeding resumed
Day 14	Non-invasive ventilation discontinued
Day 71	Discharge without gastrointestinal complications

## Data Availability

No new datasets were generated or analyzed in this study. All relevant data are included in the article.
